# Value addition and farmers: Evidence from coffee in Ethiopia

**DOI:** 10.1371/journal.pone.0273121

**Published:** 2023-01-30

**Authors:** Seneshaw Tamru, Bart Minten

**Affiliations:** 1 International Growth Center, Ethiopia; 2 International Food Policy Research Institute, Washington, DC, United States of America; Szechenyi Istvan University: Szechenyi Istvan Egyetem, HUNGARY

## Abstract

Local value-addition in developing countries is often aimed at for upgrading of agricultural value chains, since it is assumed that doing so will make farmers better off. However, transmission of the added value through the value chain and constraints to adoption of value-adding activities by farmers are not well understood. We look at this issue in the case of coffee in Ethiopia–the country’s most important export product–and value-addition in the coffee value-chain through ‘washing’ coffee, which is done in wet mills. Washed coffee is sold internationally with a significant premium compared to ‘natural’ coffee but the share of washed coffee in Ethiopia’s coffee exports has stagnated. Relying on a unique primary large-scale dataset and a combination of qualitative and quantitative methods, we examine the reasons for this puzzle. The reasons seemingly are twofold. First, labor productivity in producing red cherries, which wet mills require, is lower than for natural coffee, reducing incentives for adoption, especially for those farmers with higher opportunity costs of labor. Second, only impatient, often smaller, farmers sell red cherries, as more patient farmers use the storable dried coffee cherries as a rewarding savings instrument, given the negative real deposit rates in formal savings institutions.

## Introduction

Developing countries are typically advised to add value locally to their primary agricultural products and ‘move up’ global value chains [1–3]. This has been considered essential for them to bring their citizens out of poverty and better their living standards [4, 5]. Such beliefs are reflected, for example, by statements of the G20 heads of states: “We need policies that take full advantage of global value chains and encourage greater participation and value-addition by developing countries” [6] or by the United Nations: “GDP growth alone, based chiefly on exports of oil, minerals, and agricultural commodities with little or no processing involved, has not led to sustained poverty reduction” [7].

When food systems transform—due to urbanization, infrastructure development, food policy reform, and/or diet change—consumers are willing to pay more as a reward for quality, safe, standardized, certified, packaged, branded, ready-to-eat, and processed foods. This often implies that the share of the farmer in the final consumer price decreases [8]. [9] show in a study representing 90 percent of the global economy that the off-farm share in food expenditures makes up as much as 73 percent and that this share is strongly and positively related with rising incomes. Given these changing food expenditures with rising incomes, it is believed that agri-food systems of developing countries can capture a bigger share of the growing food consumption budget through value chain upgrading and value addition [10].

A number of value addition activities are often noted as food systems transform. We discuss four in particular: certification of standards, packaging and branding, processing, and quality. First, with transformation, there are more demands for food standards. In the beginning of the transformation process, these standards are typically imposed by the public sector but in more advanced systems, private standards typically take over [11]. One important global trend is the increasing demand for Voluntary Sustainability Standards (VSS). Consumers are willing to pay extra for produce when they are assured that products are produced and processed following adherence to ethical, safety, and environmental sustainability standards, proven by a certification of a VSS certified organization which put documentation systems in place to assure adherence to such standards [1].

Second, in traditional food systems, products are mostly sold loose, unbranded, and unpacked. In transitional and modern system, we see the emergence of packaged and branded products [8, 12]. For example, [13] showed recently in Asia the rapid increase in rice markets of branded and packaged rice, sold by wholesalers, millers, as well as modern retailers to ultimate consumers. [14] illustrated in India how branding quickly emerged in a traditional value chain. Brands develop in agricultural markets to distinguish produce from unpacked and loose products and by adding a brand value–guaranteeing consistent quality and adherence to standards–companies are able to demand higher prices for their products. This is typical for differentiation processes taking place in these transition and modern markets [8].

Third, with transformation, there is increasing consumption of processed foods, as they allow for longer storage and easier transportation and food preparation. Large investments by small and medium enterprises and large companies are therefore seen that process foods. However, this increased consumption of processed products is often associated with important health concerns, especially in the case of ultra-processed products, as shown in higher incidences of the double burden of malnutrition in low- and mid-income countries, i.e. the coexistence of high levels of undernutrition (stunting and wasting) with overnutrition (obesity and overweight) in the same country [15].

Fourth, quality and standardization become much more important in transitional and modern food systems [8]. In traditional markets, rewards to quality are relatively small and grading services by a third-party do not pay, bad and better-quality products are often mixed, or better-quality products are not produced at all [16–18]. In transitional and modern system, these quality premia rewards become much bigger (or a market for lower quality produce is simply lacking), giving producers incentives to produce that higher quality produce [8].

There are surprisingly few empirical studies that have analyzed what happens to the additional ‘value’ obtained when agricultural producers in developing countries move into higher value products and try to move up the value chain. Therefore, it is useful to obtain further insights on several important questions related to how that value is distributed along different links in the value chain, what share of the value-added accrues to farmers in low- and mid-income countries, and what constraints there may be to participation in these value-addition activities. To fill this gap, we explore the case of coffee in Ethiopia, its most important export product.

Coffee is an interesting product for such a study given its global importance and the potential it has for value-addition through different means because of its differentiated upstream markets [19]. Value can be added, for example, through specialty production [20, 21], adherence to Voluntary Sustainability Standards (VSS) [22–24], organic production practices [25], roasting [26] or single-serve coffee pods [27]. The value of coffee also depends importantly on the type of processing, i.e., whether it is processed through wet or dry processing. In wet processing, commonly known as ‘washing’, fresh red cherries are de-pulped, fermented, and washed using wet-milling machines. In the more traditional dry processing, cherries are first dried–often in the house of the farmer–and then hulled using mechanical hullers. As the investment costs in wet mills are much higher and the resultant product is perceived to be of higher quality, there are significant premiums paid for washed coffee in international markets [28, 29].

We rely on unique large-scale datasets and a combination of qualitative and quantitative methods to tackle two research questions related to value addition—through washing—in the coffee sector in Ethiopia. First, to what extent is there transmission of the quality premium—due to value addition activities—across layers of the value chain? Second, what are the constraints to the adoption of red berries—the primary product to produce washed coffee–at the farm level? There are three main findings from our research. First, we find that washed coffee from Ethiopia is being sold internationally with a substantial premium, ceteris paribus, and that this premium is largely transmitted to producers. However, we also find that only a minor share of Ethiopia’s coffee is exported as washed and that this share is not increasing over time, implying that Ethiopia is losing out on much needed foreign exchange earnings. Even if coffee farmers have access to a wet mill, they often do not sell all their coffee cherries to them, indicating likely important constraints to the adoption of washed coffee production.

Second, we find that labor productivity to produce red cherries, which are required by wet mills, is significantly lower than for dried cherries. This reduces the incentive for adopting the higher value-adding activity. This finding provides further evidence to research that shows that labor scarcity and labor costs can explain low levels of adoption of seemingly promising new agricultural technologies [30, 31]. While most of the literature on agricultural labor constraints focuses on new agricultural production technologies, we contribute to the literature by studying value-addition based on new harvesting technologies and access to alternative processing methods.

Third, only impatient farmers, who often are smaller and poorer, sell red cherries. More patient farmers use storable dried cherries as a savings instrument because of the negative real deposit rates in formal savings institutions. This finding adds to the literature highlighting the often-important interlinkages between output, input, credit, and savings markets in commodity marketing [1]. When real savings in formal institutions are not properly rewarded, farmers connected to international markets and prices may forego seemingly profitable agricultural marketing options in order to address imperfect savings and credit markets in these settings [32]. It seems that relatively richer coffee farmers often use such an option, possibly resulting in subsequent welfare differences [33].

## Coffee and processing in Ethiopia

Ethiopia is Africa’s biggest coffee producer and exporter [34]. Coffee is an important cash crop and plays a crucial role both for the national GDP and in the livelihoods of millions of people [35–37]. Approximately 15 million people directly or indirectly rely on income from the sector for their livelihood [34]. Coffee is almost exclusively a smallholding business as smallholder farmers account for 95 percent of total coffee production [36]. Ethiopian coffees are further known for their Arabica varieties with unique and much appreciated taste, giving them a significant premium in international markets. Despite these premiums, coffee yields in Ethiopia are low given the limited adoption of improved production practices [38].

Harvested coffee cherries go through several processes before the coffee can be sold in the international market. Outer layers of the coffee cherry are removed, leaving only the coffee bean surrounded by a silver skin and parchment layer, known as green coffee. Coffee is internationally traded in this form. Two different methods are used to remove these layers: wet and dry processing. Wet processing generally increases the quality of coffee [39]. Wet-processed (hereafter, washed) coffee preserves the intrinsic quality of the bean better than does dry-processed (hereafter, unwashed) coffee, with the process leading to more homogenous coffee with fewer defective beans. Washed coffee, therefore, generally is sold at significantly higher prices in international markets [29].

The wet method differs from the dry method in that the skin, pulp, and sugary mucilage layers are removed before drying using water [40]. The wet-processing is carried out with wet-mill machines where cherries are pulped immediately after harvesting, fermented in tanks, and washed in clean water to remove the mucilage. The wet parchment coffee is then dried in the sun for up to three weeks until the moisture level reaches about 11 percent [35]. For dry-processing, cherries are dried on mats or concrete floors to avoid contact with the soil, as this affects quality, usually by the farmers themselves. After drying, the outer layer of the cherries is removed by hulling in ‘dry mills’.

Coffee smallholders sell their coffee in unprocessed whole form, either as red or dried cherries. They do not directly process their coffee into washed or unwashed beans. The farmers rather sell the cherries to either wet-processors or dry-processors that, in turn, process the cherries into the respective washed or unwashed beans. In the case of washed coffee, fresh red cherries should be delivered to washing stations within 10 to 12 hours of picking; otherwise, the cherries are no longer suitable for washing [35]. In contrast, the whole dried cherries can be kept for a long period after harvest as they can be processed (hulled) into unwashed beans afterwards in the off-season. While coffee farmers do not directly process the cherries themselves, their decision to sell coffee in red or dry form determines how their coffee is going to be processed. Coffee farmers, hence, play a key role in the development and the success of the respective coffee processing sub-sectors and, therefore, in coffee value-addition in Ethiopia.

## Data and methodology

### Data

To study the benefits of washing on export and local prices, the use of wet mills, and adoption of sales of red cherries, we rely on different unique and primary large-scale datasets. The Institutional Review Board of the International Food Policy Research Institute granted approval for this study. The survey was carried out in collaboration with the Ethiopian Development Research Institute, a think tank linked to the government of Ethiopia. Consent was informed and written. The data are publicly available on the Harvard Dataverse website (see:https://dataverse.harvard.edu/dataset.xhtml?persistentId=doi:10.7910/DVN/GRIAV8).

First, a survey of 1,600 coffee producers was fielded in February 2014. We focused on those zones that produced the most coffee in the country. To select the producers for the survey sample, the zones were stratified based on the coffee variety produced as classified for export markets–Sidama, Jimma, Nekempte, Harar, and Yirgacheffe. 320 producers were interviewed in each stratum, for a total sample size of 1,600 producers across the five strata. A comprehensive household survey was implemented, including questions on demographics, assets, access to services, and income generating activities as well as specific questions related to coffee on technology adoption, production practices and labor use, and marketing of red and dried coffee cherries.

Second, we collected information on prices offered for red and dried cherries to producers from primary cooperatives and private traders in major producing areas. Price data were collected from 12 major coffee producing zones in the country in 2013. In each zone, the three top producing woredas were selected and all primary cooperatives and private millers in each selected woreda were visited. For each trader or cooperative visited, we inquired if they had kept records on transaction prices, quantities, and total amounts paid out over the last eight years and, if so, in what form these records were kept, i.e., receipts or a “record book”. In the case where a transaction record book was maintained, we photocopied the book in the nearest town, and those data were subsequently entered into a database. Using this method, we were able to collect price information for almost 150,000 transactions of red and dried cherries from 89 cooperatives and 138 private traders. Moreover, a survey of the primary cooperative unions from which we obtained these prices was conducted in July 2014.

Third, a database of coffee export transactions is maintained by the Ministry of Trade. This dataset was obtained for the period July 2006 to June 2014. An important aspect in coffee exports is quality. Quality assessments for exports are conducted by the Coffee Liquoring Unit (CLU) to ensure that the coffee meets export standards. A quality inspection sheet is prepared by the CLU and is attached to the lot to be exported. These quality indicators, which includes washing as well as others quality characteristics such as certification and origin, are part of the coffee export transactions dataset. We also obtained a list of private commercial coffee farms (with cultivated areas of 40 hectares and above) from the association of commercial coffee farms. This information was integrated into the dataset for analysis as well.

There is one important caveat with the data collection process. While we were able to gather a unique combination of datasets, the empirical analysis relies on the estimation of average effects of washing on coffee prices based on different samples and slightly different time periods. It could be argued that the estimated coefficients therefore are not directly comparable over different levels of the value chain. To address this potential problem, we focus our analysis on the results of the most recent years of the study period, i.e., closest to the period in 2014 when the comprehensive coffee household survey was fielded.

### Methodology

In order to address the research questions, we use a mix of different methodologies. First, we test if there are premiums for washed coffee at the export level and for red cherries at the producer level. To do so, we employ a hedonic pricing model [41, 42]. This method allows for the valuation of the different attributes of an item (in our case coffee) on the (economic) value of the item. Hence, the coffee price is regressed against several coffee attributes that could affect its value. A model of the following form is specified (see e.g., Bajari et al. 2012):

$$P_{it}=\beta_{k}X_{it}^{k}+V_{i}+U_{it}$$ (1)

where P_it_ is the coffee price in US cents per kg received by firm/household i at time t. $X_{it}^{k}$ is a K-dimensional row vector of time-varying different attributes of coffee. *β*_*k*_ is a K-dimensional column vector of parameters. *V*_*i*_ is firm-specific effect while *U*_*it*_ is an idiosyncratic error term.

Second, we model factors associated with the decision to sell red cherries and the amount of coffee sales in red cherry form. A substantial proportion of coffee producers, for several possible reasons, are observed with zero sales of coffee in red cherry form. The particular interpretation given to zero observations has a crucial bearing on the estimation approach adopted. Such a relationship can potentially be estimated with limited dependent variables methodologies in the form of a Tobit model [43] or the Generalized Tobit model proposed by [44]. Nevertheless, both models rely on restrictive underlying assumptions. While the Tobit model treats all zero values as results of a corner solution and attributes them only to economic factors, the Heckman selection model attributes all zeros to non-economic considerations. In our analysis, we employ the Double Hurdle (DH) model. The DH technique compromises between the two previous techniques. It assumes that producers are faced with two hurdles and make two subsequent decisions: (1) whether or not to sell coffee in red cherry form; and (2) given the first decision, how much red cherries to sell [45–47]. The technique relies on two crucial assumptions: the level of independence between the residuals in the two decisions and dominance, i.e., whether the participation decision dominates the quantity decision. As right-hand side variables, we use household characteristics, assets (including means of transport), measures of labor costs and availability (wages, labor in household, dependency ratio), and location with respect to relevant services (distance to paved roads, banks, and mills).

Third, we analyze the effects of selling coffee in red cherry form on total labor use, labor productivity, and coffee income. To do so, we categorize farmers into two groups: farmers whose share of red cherry sales is 0 (‘untreated’) and farmers whose proportion of red cherry sales is greater than 0 (‘treated’). Given that farmers could sell both red and dried cherries, we consider the proportion of red cherry sales as the treatment variable that ranges from zero (no red cherry sales) to 100 (all coffee sales in red cherry form). Following [48], such continuous levels of treatments can be modeled with a Dose-Response Function (DRF) approach. DRFs have been widely used in economics in a number of settings in agriculture [49], nutrition [50], trade [51], and poverty analysis [52]. The most commonly applied DRF approach is the one introduced by [48] and its variants (such as [53]) which is based on the generalized propensity score (GPS). The approach, however, relies on a rather restrictive assumption, i.e., normality (or a mixture of normal distributions) of the treatment variable, which has been identified as a major drawback [54, 55]. Other studies [56] also recognize a spike at zero and propose a nonparametric two-stage matching estimation even though their method only works under assumption of conditional independence. In this paper, we employ [57] recently modified version of the DRF, which does not require a full normality assumption and is also suitable when the treatment variable has concentration at zero.

Following [57], the DRF can be specified as follows. Consider a random sample of units indexed i = 1,…,N and let *x*_*i*_ = (*x*_1*i*_, *x*_2*i*_,…, *x*_*Mi*_) be a row vector of M exogenous explanatory variables (observable confounders). Define *w*_*i*_ as a treatment indicator variable, taking 1 when treated and 0 when untreated. Let t denote the continuous treatment variable of interest, the dosage (in our case, proportion of red cherry sales) takes values in *t* ∈ *τ*, where *τ* is an interval [0,* *100]. Also, define h(*t*_*i*_) as a general derivable function of *t*_*i*_. Suppose *Y*_*i*_(t) represent two mutually exclusive outcomes of interest: *Y*_*i*1_, when unit i is treated (t > 0), and *Y*_*i*0_, when the unit is not treated (t = 0). Assume a data generation process that results in the two mutually exclusive outcome variables of this form:

$$\left\{ \begin{array}{c} w=1:\;y_{1}=u_{1}+g_{1}(x)+h(t)+e_{1}\\ w=0:\;y_{0}=u_{0}+g_{0}(x)+e_{0} \end{array}\right.$$ (2)

Where *g*_*i*_(*x*) is unit i’s responses to a vector of explanatory variables *x*_*i*_ when the units are treated or untreated. *u*_*i*_’s are two scalars, and *e*_1_ and *e*_0_ are random variables with unconditional mean and constant variance. Given these, we can define the average treatment effect (ATE| x, t) = E (*y*_1_−*y*_0_)|x, t).

Assuming conditional mean independence and given iterated expectations, the DRF can be estimated by taking the average of the following equations:

$$\left\{ \begin{array}{l} ATE=p(w=1)(\mu +{\bar{X}}_{t>0}\delta +{\bar{h}}_{t>0})+p(w=0)(\mu +{\bar{X}}_{t=0}\delta )\\ \;ATET=\mu +{\bar{X}}_{t>0}\delta +{\bar{h}}_{t>0}\\ \;ATENT=\mu +{\bar{X}}_{t=0}\delta \end{array}\right.$$ (3)

Where ATE is the unconditional average treatment effect, ATET is the average treatment on the treated, ATENT is the average treatment on the untreated, p(·) is a probability, and h_t>0_ is the average of the response function when t>0. Assuming a linear-in-parameters parametric form for *g*_*i*_(*x*) = *xδ*_*i*_, δ is a vector of coefficients of explanatory variables, and μ = *u*_1_−*u*_0_, and δ = δ_1_ − δ_0_. Consequently, the DRF is estimated by averaging ATE(x, t) over x. It is, therefore, a function of the treatment intensity [57].

## Results

### Quality premiums for washing

#### At export level

**Fig 1:**
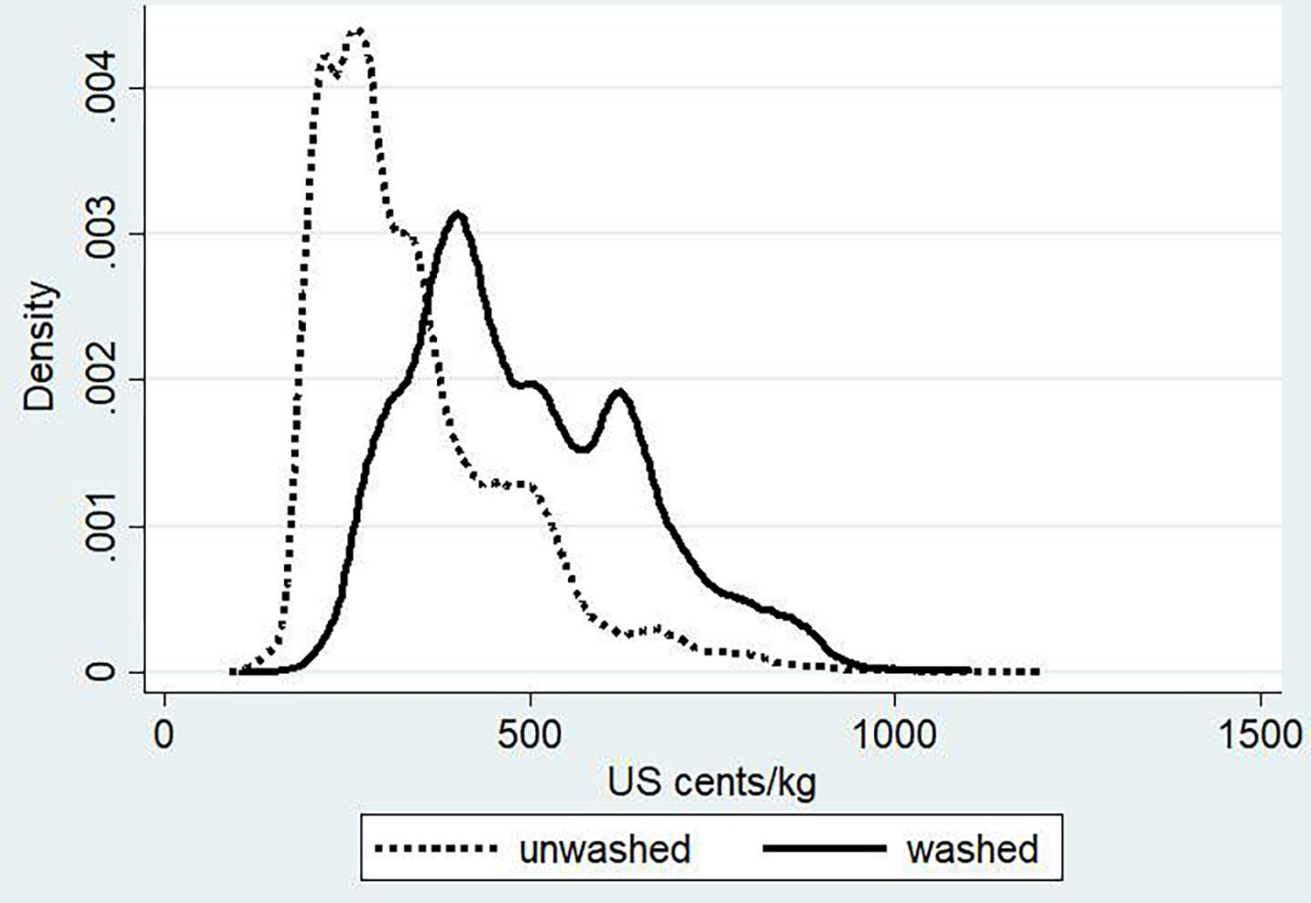
Price benefits of washed coffee in Ethiopia–density function of washed versus unwashed coffee prices at the export level. Source: Authors’ calculation based on ERCA data (2008–2014).

Simply using observed prices of each exported lot expressed in US cents per kg, Fig 1 illustrates the size of the washed coffee premium over the period 2006 to 2014. The density function of prices of washed coffee is distinctively to the right of natural coffee, indicating significant premiums for washing at the export level. The average price difference over that period amounts to 152 US cents per kg, statistically different when measured with a t-test (t = -91.13; Pr(|T|>|t|) = 0.00). However, this simple price comparison masks other factors that might be associated with the washing premiums. To get at the additional value of washing on top of other variables, a multi-variate regression framework is required.

Following the methodology outlined in Section 3, we regress export prices of clean green beans in US cents per kg on processing method (washed or unwashed), whether coffee is organic or not, type of exporters, and year-monthly dummy variables. In addition, we also control for the geographic origin of coffee which is an important quality consideration, as it is strongly related to taste. In our analysis, we distinguish between Sidama, Jimma, Wollega (Nekempt), Yirgacheffe, and Harar coffees. [58] associates tastes and regions as follows: spicy for Sidama, fruity for Wollega (Nekempt), floral for Yirgacheffe, winy for Jimma, and mocha for Harar. Generally, coffee originating from Yirgacheffe and Harar coffee are sold, ceteris paribus, at higher prices than the Sidama coffee [29]. In contrast, coffee produced in Wollega or Nekemte and Jimma are valued less than the coffee originating from Sidama.

We present four specifications in Table 1. In the first specification, we report the results of a pooled regression. In this case, washing raises the price of exports significantly by 99 US cents per kg. When we use an exporter fixed effect model in order to control for observables and un-observables at that level and include time dummies but no other controls (Specification 2), we find that washing raises prices significantly by 112 US cents per kg. When we run the exporter fixed effect model with a number of other controls for the whole period (the third specification), washing shows a slightly lower premium of 93 US cents per kg, *ceteris paribus*, indicating that washing is especially associated with coffee grown in highly appreciated origins of Yirgacheffe and Sidama [29]. We are specifically interested in the premiums in most recent years of the dataset. We therefore restrict the sample to the years 2012 and 2013. In this fourth specification, we find that washing raises coffee prices by 102 US cents per kg. For all the specifications, the washing dummy is highly significant and the quality premium for washing adds a premium compared to natural coffee of between 28 and 33 percent, depending on the specification.

**Table 1:** Associates of coffee prices in US cents per kg at export level, 2006–2013.

Variables	Specification 1		Specification 2		Specification 3		Specification 4	
	Coef.	t-value	Coef.	z-value	Coef.	z-value	Coef.	z-value
Dependent variable: unit price (US cents per kg)								
Washed	99.09***	68.49	111.98***	108.85	93.12***	21.63	102.30 ***	15.60
Controls:								
Organic	yes		no		yes		yes	
Exporter type	yes		-	-	-		-	
Destination market	yes		no	-	yes		yes	
Local origin	yes		no		yes		yes	
Year-month of export	yes		yes		yes		yes	
Intercept	168.73***	59.77	211.13***	79.64	197.11 ***	21.50	244.11***	48.62
Regression method	Pooled		Fixed-effect exporter level		Fixed-effect exporter level		Fixed-effect exporter level	
Period	2006–2013		2006–2013		2006–2013		2012–2013	
Number of observations	30,638		30,625		30,599		10,207	
Number of groups	-		295		294		209	
R-square overall	0.77		0.59		0.70		0.60	
R-square within	-		0.64		0.71		0.64	
R-square between	-		0.40		0.48		0.27	

Source: Authors’ calculations based on data from the Ministry of Trade

Note: Standard errors clustered at exporter level

*** p<0.01

** p<0.05

* p<0.1

#### At producer level

**Fig 2:**
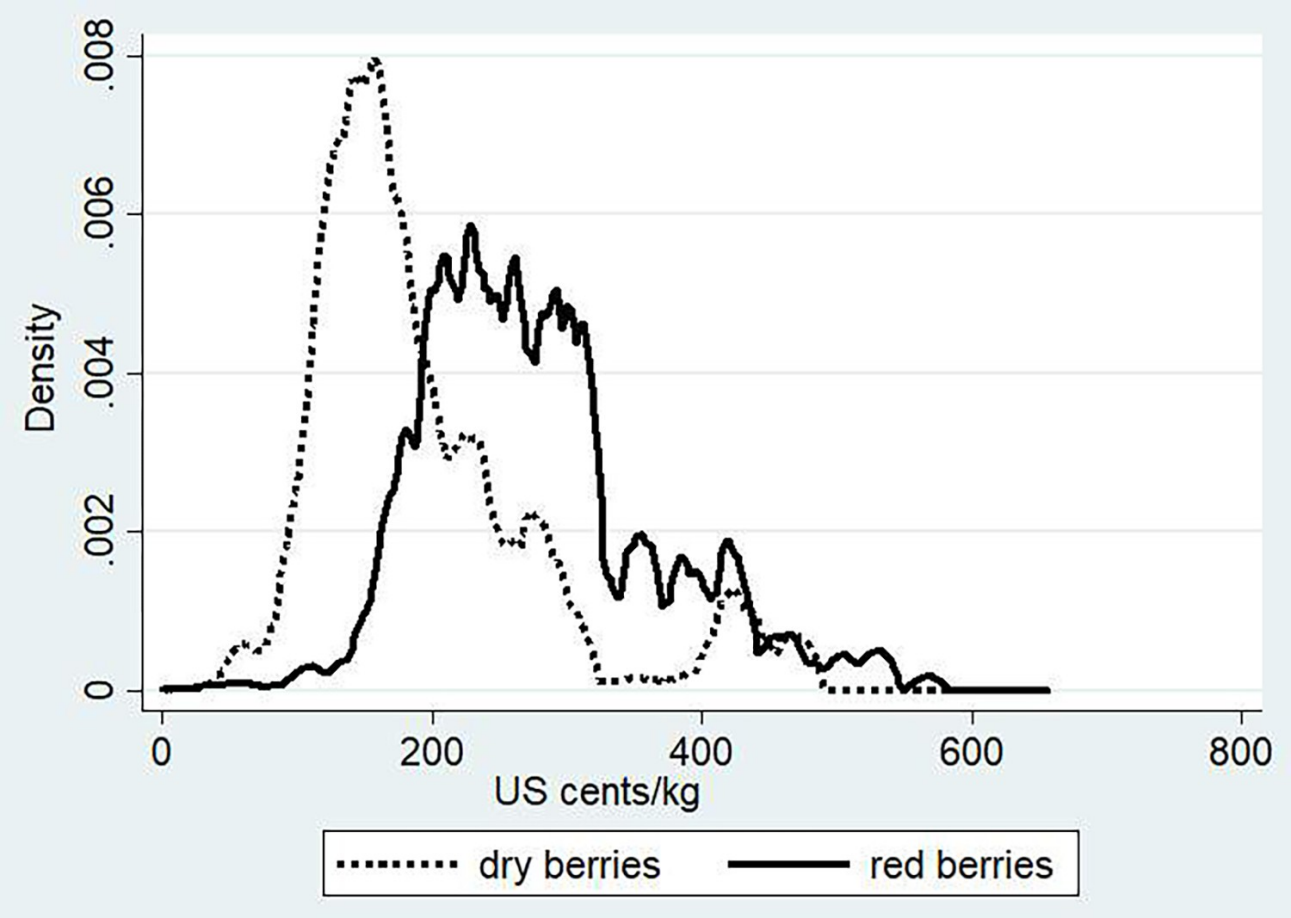
Density function for dry and red coffee cherries—in clean green coffee equivalent—based on producer prices time series, US cents per kg. Source: Authors’ calculation based on data collected from cooperatives (2004–2014).

To analyze at the producer level the quality premiums associated with washing and related sales of red cherries, we use a time series of producer prices over an eight-year period from 2006 to 2013 that was collected from cooperatives and private traders. For each buyer, information was obtained on all sales transactions over that period. We use as our dependent variable prices in Birr/kg and then use prices converted to US cents per kg and clean green coffee to make prices comparable between producer and export levels. Price density functions on prices for red and dried cherries are shown in Fig 2. When we consider the appropriate conversion factors, where the typical conversion ratios used of red and dried cherries to green clean coffee are 1:6 and 1:2 respectively [59–61], a picture emerges with effective positive price premiums being paid for red cherries, as illustrated by a price density function in US cents/kg to the right of dried cherries.

The results of different regression specifications–following a similar set-up as at the export level–are presented in Table 2. We only present results of regressions where prices are expressed in US cents per kg of clean green coffee. We first run a pooled regression where we regress the price of cherries on the form of the cherry (red cherries or dried cherries), VSS certification, type of buyer, an origin variable, and month-yearly dummies. In this specification, we find that selling in red cherries raises producer prices by 78 US cents per kg. In a second specification, we run a fixed effect by buyer model and control only for monthly and yearly dummies. In this specification washing raises prices by 68 US cents per kg, a lower amount than in the first specification. In a third specification, on top of the second specification, we include certified buyers and origin dummies as well. In this model, we find that washing raises the producer price by 68 US cents per kg. Finally, we focus only on the most recent period in our dataset (Specification 4). The premium is lower in this specification (at 56 US cents per kg), but still in the same order of magnitude as at the exporter level.

**Table 2:** Associates of producer prices of red cherries converted to clean green beans based on prices collected from buyers.

Variables	Specification 1		Specification 2		Specification 3		Specification 4	
	Coef.	t-value	Coef.	t-value	Coef.	t-value	Coef.	t-value
Dependent variable: unit price (US cents per lb)								
Washed	77.87***	40.32	67.51***	3.89	68.47***	3.80	56.17***	3.37
Controls:								
Certified buyer	yes		no		yes		yes	
Type of buyer (private or cooperative	yes		-		-		-	
Origin	yes		-		-		-	
Year-month of purchase	yes		yes		yes		yes	
Intercept	-14.51***	-5.97	-11.00	0.67	-14.27	-0.76	22.63	1.52
Regression method	Pooled		Fixed-effect buyer level		Fixed-effect buyer level		Fixed-effect buyer level	
Period	2006–2013		2006–2013		2006–2013		2012–2013	
Number of observations	146,905		146,905		146,905		33,862	
Number of groups	-		243		243		241	
R-square overall	0.85		0.77		0.78		0.76	
R-square within	-		0.85		0.85		0.85	
R-square between	-		0.63		0.63		0.73	

Source: Authors’ calculations based on producer prices collected from primary cooperatives and private traders.

Note: Standard errors clustered at buyer level

*** p<0.01

** p<0.05

* p<0.1

One issue with using this method in the estimation of the washing premium is that buyers may pay the full price only after they have secured a buyer for the lot and have been paid [38]. Analysis of prices recorded at the time of the initial transaction might therefore potentially underestimate the benefits of washing [29]. To understand to what extent second payments are raising producer prices paid for red cherries, we rely on the reported initial prices and the second payment for every coffee transaction at the household level during the year 2013. This information was collected during the farm survey done in 2014. Following the model of Section 3, we regress prices for coffee expressed in clean green beans on the form of the cherry, certification of the buyer, place of sales, month of sales, and woreda dummies. We run two different specifications (Table 3). In specification 1, we run a pooled regression and find that red cherries have a premium of 46 US cents per kg. In a second specification, we run a fixed effect model at the household level in order to control for unobservables at that level. Compared to selling dried cherries, under this specification selling red cherries provides a premium of 37 US cents per kg. Again, selling cherries in the red form leads to a significantly higher price in both specifications.

**Table 3:** Associates of producer prices of red cherries converted to clean green beans based on prices collected from producers.

Variables	Specification 1		Specification 2	
	Coef.	t-value	Coef.	t-value
Dependent variable: unit price (US cents per kg)				
Red cherries	45.64***	3.67	36.84***	4.21
Controls:				
Certified buyer	Yes		-	
Place of sale	Yes		yes	
Monthly dummies	Yes		yes	
Woreda dummies	Yes		-	
Intercept	142.51***	7.48	207.92***	12.20
Regression method	Pooled		Fixed effect household level	
Number of observations	3,353		3,353	
Number of groups	-		1,461	
R-square overall	0.82		0.00	
R-square within	-		0.10	
R-square between	-		0.02	

Source: Authors’ calculations based on ESSP’s coffee producer survey 2014.

Note: Standard errors clustered at the kebele level

*** p<0.01

** p<0.05

* p<0.1

We therefore find that there are significant premiums at both the export and producer levels for washing coffee and for selling red cherries and that price premiums for washed coffee from exporters are transmitted to farmers. Similar results on the benefits of sales of red cherries have been documented in other countries [62]. In the next sections, we assess to what extent these higher premiums are associated with the use of wet mills and with adoption decisions by coffee producers to sell red cherries.

### Use of wet mills

We first assess how access to and investments in wet mills are changing. In the last decade, a large number of wet mills have been started up in Ethiopia. In the community survey, focus groups were asked to report on the number of mills in their community and indicate when they were started or acquired by the current owner. Of all the wet mills in the kebele at the time of the survey, only one-third had been in place ten years earlier. Most of owners had started their mills– 85 percent of all the wet mills were built by the current owner.

These increasing investments and easier access to wet mills is confirmed by other data. Farmers were asked if they had the option to sell red cherries at the time of the survey and ten years earlier. We note significant changes in access to wet mills (Table 4). While only 15 percent of the farmers reported that they could sell red cherries ten years before the survey, this had increased to 42 percent of the farmers at the time of the survey. We also see an increase in the number of mills in the kebele and a decrease in the distances that farmers have to travel to those mills. The density of wet mills within a kebele increased significantly as well. While 7 percent of the surveyed communities had access to two wet mills or more in the community ten years prior to the coffee survey, this had increased to 25 percent of the communities at the time of the survey.

**Table 4:** Access and use of wet mills by coffee farmers.

	Unit	At time of survey	Ten years earlier	z or t-value
*A*. *Access to wet mills*				
Farmer has the option to sell red cherries	% of farmers	42.5	14.8	3.19 ***
Travel time to nearest wet mill	minutes	89.4	110.4	-9.82 ***
Travel time to second nearest wet mill	minutes	103.9	123.2	-8.63 ***
Average number of wet mills in the kebele	number	0.7	0.3	3.86 ***
Communities with:				
0 wet mills	share	66.3	81.3	
1 wet mill	share	8.8	11.3	
2 wet mills	share	17.5	3.8	
3 or more wet mills	share	7.5	3.8	
*B*. *Use of wet mills*				
Share of coffee sold as red cherries	%	19.3	13.5	10.98 ***
Travel time to sell red cherries	minutes	30.2	41.9	-12.76 ***
*C*. *Market regulation at the time of the survey*				
Farmers sold more red cherries than they wanted because they were obliged to do so by the authorities	% yes	6.5		

Source: Authors’ calculations based on ESSP’s coffee producer survey 2014

Despite these increasing options to sell to wet mills and premiums for the sales of red cherries for producers, we note that adoption of sales of red cherries to wet mills is much lower than accessibility to wet mills would suggest. First, Table 4 shows that the share of coffee farmers that sell to wet mills has significantly increased over the ten years prior to the survey–increasing on average from 13 to 19 percent of all the coffee sold, a 50 percent increase. However, the share of coffee farmers selling to wet mills is significantly below potential. Second, the government does not allow in some regions the sales of dried cherries during the period of marketing of red cherries, so as to stimulate the output of red cherries. While the number of farmers that reported selling more red cherries than they wanted to is rather limited at 6 percent of all coffee farmers, these regulations are indicative of resistance by farmers to selling their coffee in the form of red cherries. Third, examining the share of washed coffee exported from Ethiopia over the period 2006 to 2013, it is found that the share of washed coffee in total export has not changed over this period. While the share fluctuates from year to year, it has stayed, on average, around 30 percent of all coffee exports. The figure also illustrates the considerable seasonality in exports of red cherries compared to dried ones. In line with the red berry harvest season, the share of coffee exports from Ethiopia made up of washed coffee is substantially higher at the end of the year.

Therefore, we note, on the one hand, overall increasing access and investments in wet mills in rural areas in Ethiopia and, on the other hand, that the share of washed coffee in exports is not increasing to the same extent that one would have expected with the increasing options for selling red cherries to wet mills available to coffee farmers. This is not unique to Ethiopia. [63] also documented a case where coffee farmers in Rwanda were reluctant to sell in red berries even when they had the possibility to do so (this consequently led to lower levels of washed coffee production in the country with the washing stations operating below capacity). The farmers rather preferred to sell in what the authors call ‘ordinary coffee’ (i.e., dried berries). This then begs the question why. We discuss this issue in the next section.

### Household adoption and constraints

#### Sales of red cherries by coffee farmers

We start by assessing the factors that are associated with red cherry sales vis-à-vis dried ones. As explained in the methodology section, we rely on a double-hurdle methodology. Table 5 presents descriptive statistics of variables used in the model as well as the coefficients and significance of factors possibly associated with the decision and the amount of red cherry sales. Estimates are presented for the first and second hurdles, and for Average Partial Effects (APE). Some interesting insights emerge from the results of this regression.

**Table 5:** Associates of sales in red cherries, double-hurdle model.

Variables	Unit			Results from double hurdle model					
		Summary statistics^#^		Decision to sell red cherries (mfx)		Quantity of red cherries sold (mfx)		Average partial effect (Cragg)	
		mean	sd	mfx	z-value	mfx	z-value	mfx	z-value
Coffee sales of red cherries	percent	17.24	26.33						
Daily wage rate	Birr/person days	27.56	12.43	-0.66***	-4.43	-6.60**	-2.42	-2.16**	-2.46
Time preference (default = time neutral)									
Time patient	yes = 1	0.17	0.38	0.23	1.63	0.79	0.23	0.25	0.23
Time impatient	yes = 1	0.51	0.50	0.19*	1.72	15.12***	4.87	4.72***	5.05
Total active labor in HH	log(number)	1.62	1.63	0.34*	1.72	0.45	0.09	0.14	0.08
Ratio of dependents	percent	48.69	20.81	0.00	0.99	0.02	0.22	0.00	0.2
Total household assets	log(Birr)	16,870	21,504	-0.22***	-5.22	-0.84	-0.87	-0.26	-0.82
Characteristics of household head									
Gender	male = 1	0.94	0.23	0.16	0.63	7.88	1.28	2.46	1.17
Age	log(number)	44.84	14.57	0.54***	2.90	5.47	1.25	1.71	1.21
Education (dummy)	educated = 1	0.66	0.48	0.35***	2.92	-1.99	-0.54	-0.62	-0.55
Additional controls									
Other household characteristics^##^				yes		yes		yes	
Distance to nearest services^###^				yes		yes		yes	
Coffee regions				yes		yes		yes	
Intercept				3.87***	3.85	24.54***	21.61		
Observations						1,198			
Sigma						24.54***			
Log pseudo-likelihood						-2601.33***			
Wald Chi^2^()						5475.69***			

Source: Authors’ calculations based on ESSP’s coffee producer survey 2014

*** p<0.01

** p<0.05

* p<0.1; standard errors for APE were calculated through bootstrapping with 200 replications using the ‘craggit’ stata routine (Burke 2009)

# All variables under statistics are in levels (i.e., non-logarithms).

## including religion, marital status of head, and wealth indicators.

### distance to: nearest formal saving institution, nearest all weather road, nearest dry hulling machine

First, a number of variables indicative of higher opportunity cost of labor show significant negative associations with the adoption of the sales of red cherries. For example, rural daily wages are negatively related to both the decision and amount of red cherry sales. The higher the rural wage levels, the lower the likelihood and quantity of selling coffee in red cherry form–a Birr increment in daily wage rate would reduce the volume of red cherry sales by seven kilograms. The likelihood of selling coffee in red cherry form also increases with the number of active working-age members in a given household. Farmers with more active working-age members are more likely to sell red cherries compared to households with a lower number of working-age members (i.e., older than 15 and younger than 65 years). Richer coffee farmers (measured by the value of total assets) are less likely to sell in red cherry form. On the other hand, households headed by a person with some level of education are more likely to sell their coffee in red cherry form compared to households with uneducated heads. The volume of sales, however, does not seem to be affected by education level.

Second, the decision and amount of red cherry sales are also significantly associated with the time preferences of the farmers. To categorize the farmers into time preference categories, we adopted the methodology used by, for example, [64, 65]. In this method, farmers are asked hypothetical questions on their preference between a smaller immediate payment and a larger later payment over the subsequent month. Farmers were asked to choose between an immediate Birr 800 payment and a range of alternative options for a payment after a month. The payments after a month ranged between Birr 700 and linearly increased by Birr 100 up to the highest option of Birr 1500. Based on farmers’ replies, we then used the respective discount rates to classify the farmers into three distinct time preference categories: time patient, time neutral, and time impatient. We find that coffee farmers with higher discount rates, i.e., the impatient ones, are more likely to sell their coffee in red cherry form compared to time-neutral farmers. The volume of red cherry sales is also associated with impatience. Impatient farmers sell about 15 kg more red cherry coffee compared to time-neutral farmers.

### Labor productivity

Detailed information on labor use by adults and children (male and female) during different production and processing activities were collected in the coffee producer surveys, including information on tree management, mulching, tilling/hoeing, manure and compost application, weeding, chemical fertilizer/herbicide/pesticide applications, harvesting and post-harvesting activities. We use that information to get at different measures of labor productivity.

**Fig 3:**
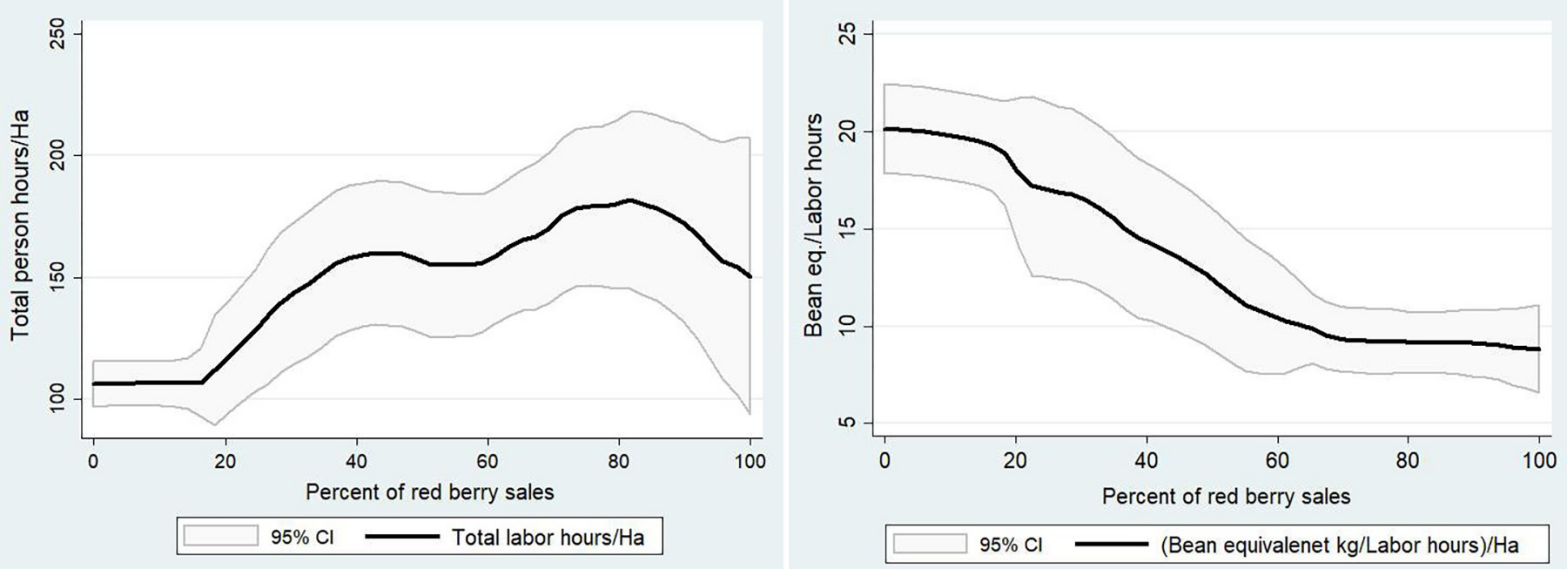
Relationship between percent of red cherry sales and labor use per hectare and labor productivity of producer. Source: Authors’ computation based on ESSP’s coffee survey.

We first present two non-parametric regressions (Fig 3). In the first one, we relate total labor use per hectare–measured in person hours–with the percentage of red cherry sales in total coffee sales. We note overall that the higher the share for red cherries the higher the labor use per hectare. In the second graph (on the right), we look at the association of the green bean equivalent produced per hour of labor. The graph suggests a strong negative relationship between share of red cherry sales and labor productivity, i.e., the production of red cherries requiring significant more labor per kg of bean than dried cherries. While red cherries are rewarded more in the market, the difference in output prices does not seem to make up for the differences in labor required.

To improve on these suggestive relationships, we implement the Dose-Response Function (DRF) approach described under the methodology section. We focus on estimating the average treatment effect (ATE) of selling coffee as red cherries. Panel A, B, and C of Table 6 present the summary results for different outcome variables. The first set of columns displays estimates of the average ATE, but we also show the responses of the different outcome variables to different doses (proportion of red cherry sales), i.e., at 10, 25, 50, 75, and 90 percent levels.

**Table 6:** Comparison of labor use, marketing costs, and labor productivity, expressed in kilograms of clean coffee per hour worked.

	Outcome variables	ATE (% of red cherry sales)–Dose Response Function				ATE evaluated at *X* percentage level of treatment				
		Coeffi-cient	t-value	R^2^	Observ-ations	10	25	50	75	90
Panel A	Income per hectare	0.302***	2.72	0.20	1,096	0.096	0.055	0.098	0.195	0.239
Panel B	Labor hours per hectare:									
	Overall	0.261**	2.17	0.29	1,331	0.248	0.182	0.192	0.181	0.084
	Harvesting activities	0.518***	2.86	0.24	1,331	0.306	0.176	0.365	0.593	0.523
	Post-harvest activities	-0.384**	-1.99	0.18	1,141	-0.345	-0.338	-0.644	-0.995	-1.059
	Marketing costs per kg	0.361**	1.96	0.22	1,331	0.109	0.408	0.532	0.462	0.459
Panel C	Labor productivity:									
	Production [green bean] per labor-hour	-0.179**	-2.27	0.21	1,185	0.026	-0.050	-0.101	-0.108	-0.114
	Income per labor-hour, Birr	-0.182*	-1.90	0.18	966	-0.032	-0.112	-0.009	-0.042	-0.333

Source: Authors’ calculations based on ESSP’s coffee producer survey 2014.

Note

*** p<0.01

** p<0.05

* p<0.1

Treatment variable endogeneity is tested using Stata’s ’estat endogenous’ command (after ’effects’). No evidence of endogeneity was found.

Panel A presents outcomes of the DRF estimates of income per hectare. The overall ATE estimate (second column of Table 6) shows that, compared to control farmers, who have zero percent red cherry sales, treated farmers, who have greater than zero percent red cherry sales, on average earn 30 percent higher coffee income per hectare. The ATE evaluated at different levels of treatment also reveals that coffee income generally rises for farmers that sell a larger proportion of their coffee in red cherry form. Looking at the ATE evaluated at 10 percent red cherry sales, coffee income for treated farmers is 10 percent larger compared to that of control farmers, while this increases to as high as 24 percent for 90 percent red cherry sales.

Under Panel B, we compare differences in labor use for different proportions of red cherry sales. We consider four outcome variables. For overall labor (non-marketing) use that includes labor used for all activities, including for tilling, weeding, compost use, harvesting, and post-harvest activities, farmers who sell their coffee in red cherry form engage about 26 percent more person-hours per hectare than those farmers who sell no red cherries. While we see a declining rate of increment across higher points of treatment intensity, the overall labor use is still larger for treated farmers than for the controls. The results overall indicate that producing red cherries for the wet processing method is more labor intensive. We explore below several contributing factors.

Farmers who sell their coffee in red cherry form use substantially more labor-hours for harvesting compared to those who do not sell red cherries. Estimates from the DRF indicate that coffee farmers that sell their coffee in red cherry form on average use 52 percent more labor per hectare compared to farmers that do not sell red cherries. This is seemingly linked to the care required during harvesting because wet mills require properly ripened cherries. ATEs evaluated at different level of treatment display similar level of harvesting labor use by treated farmers compared to the control.

On the other hand, labor use for post-harvest activities, i.e., transportation, storage, drying, etc., is substantially lower for treated farmers than for the control farmers–counteracting the positive effect of harvesting labor in overall labor use. Compared to farmers who sell all their coffee in dried form, farmers who sell at least some proportion of their coffee in red cherry form use about 38 percent less labor for post-harvest activities. Furthermore, as can be seen from the ATEs evaluated at different treatment intensity, the difference in harvesting labor use between the treated and control farmers gets even lower as treatment intensity increases. However, it is to be noted that post-harvest activities are only a minor part of overall labor use in coffee activities.

DRF estimates were also done for marketing costs, as measured by transport cost incurred. We note again that there are substantial differences between the treated and control farmers. The ATE estimates at all levels of treatment intensity show that treated farmers face larger marketing cost per kg than the control farmers. The results indicate that, on average, red cherry coffee selling farmers incur 36 percent more transport cost than those of dry cherry selling farmers. This might be due to the perishable nature of red cherries where red cherry sellers have to more frequently travel to red cherry market centers for timely delivery of red cherries to washing stations.

Finally, Panel C in Table 6 shows the results of labor productivity measures. We look at labor productivity in kilogram of clean bean equivalent per hour worked and in coffee income per hour worked. Estimates from the ATE show that labor productivity as measured by bean equivalent per hour worked is found to be lower by about 18 percent for households that sell in red cherry form. Similarly, when measured by coffee income per labor hour, coffee farmers selling their coffee in red cherry form face 18 percent lower income per labor hour compared to those famers selling their coffees all in dried form. The results are consistent for different treatment intensity, albeit with some fluctuation.

### Savings

Farmers were asked to indicate why they preferred selling in dried versus red cherry form. A number of options that were suggested during the pre-testing of the questionnaires were presented to them, including using dried cherries as a savings mechanism; bad quality, e.g., picked off the ground, so that the cherries cannot be sold as red; late ripening, so beans were not suitable to be sold during the red cherry selling season; not enough buyers for red cherries; cherries harvested early out of fear of theft, resulting in cherries being therefore not suitable for processing in wet mills; and lack of labor for red cherry harvesting. Multiple answers were allowed for. The results in Table 6 show that an overwhelming majority of the farmers (90 percent) indicated that they used the dried cherries as a savings instrument. Other reasons that were mentioned included bad quality and late ripening, both of which were mentioned by 16 percent of the farmers. Lack of buyers and of labor and early harvest because of fear of theft were relatively less important. Famers were also asked to indicate if they agreed or disagreed with the statement that “I prefer selling coffee in dried form instead of red cherries because I can spread out my income that way (it is a way of saving)”. Three-quarters of the coffee farmers agreed, again indicating the importance of dried cherries as a savings instrument.

Questions were also asked on the availability and use of formal saving institutions in the kebele. About one-third of the farmers mentioned that savings and credit associations were available in their community (Table 7). This compares to 16 percent for banks and micro-finance institutions (MFI). Even though farmers have access to these savings institutions, only half of them with access stated that they used the credit and savings associations present in the kebele for a total of 16 percent of the coffee farmers. The percentage that used banks and MFIs when they are available is significantly higher. The fact that people reported dried cherries as a savings instrument in the presence of these formal savings institutions leads us to compare the rate of return between these two forms of savings.

**Table 7:** Use of dried coffee cherries as a savings instrument, descriptive statistics.

	Unit	At time of survey
Self-reported reasons for selling dried cherries instead of red ones (multiple answers possible)		
Using the dried form as a saving mechanism	% of farmers	90.0
Bad quality, e.g., picked from the ground	% of farmers	16.3
Late ripening and not suitable to sell them as red cherries	% of farmers	16.6
Not enough buyers of red cherries	% of farmers	5.7
Harvest early because of fear of theft	% of farmers	3.3
Lack of labor for timely red cherry harvesting	% of farmers	3.4
Agreement with “I prefer selling coffee in dried form instead of red cherries because I can spread out my income that way (it is a way of saving).		
"Yes, I agree"	% of farmers	75.7
"No, I disagree"	% of farmers	19.2
"It depends"	% of farmers	4.7
"I don’t know"	% of farmers	0.4
**Access and use of formal saving institutions**		
Is this form of savings available in the kebele?		
Savings & credit association	% yes	34.0
Bank/MFI	% yes	16.0
If not available, how far is the closest one?		
Savings & credit association	kilometers	17.3
Bank/MFI	kilometers	20.0
If available, do you use this savings form?		
Savings & credit association	% yes	16.2
Bank/MFI	% yes	18.4

Source: Authors’ calculations based on ESSP’s coffee producer survey 2014

We do so by looking at seasonal differences in coffee prices and comparing that seasonal amplitude with interest rates received in formal saving accounts. In particular, we make use of the fact that the red and dry cherries have different selling seasons, i.e., red cherries are sold from August to December, while dry cherries are sold from January to July. To get at the seasonal amplitude, we consider the price received for coffee in May (the main sales month for dried cherries) compared to November (the main sales month for red cherries). This November-to-May comparison is done for the last 16 years based on price data collected by the Central Statistical Agency (CSA) in the main coffee producing regions. S1 Fig shows that coffee beans sold in May get, on average, a substantially higher price than coffees sold in November the year before. This difference was especially large in the first part of the period looked at, because of high overall inflation as well as increasing international coffee prices. On average, coffee prices in May were 19 percent higher compared to coffee sold in November over the whole period considered. This compares to an average 2.5 to 3.5 percent interest rate given in local banks over that period, indicative of highly negative deposit rates given the prevailing inflation in the country [66, 67]. If farmers want to save–an important issue in Ethiopia given the often-seasonal stress noted in these rural communities [37, 68]–the data show that keeping dried coffee is a more rewarding savings instrument than saving in formal savings institutions.

## Discussion and conclusions

Local value-addition in developing countries is often aimed at upgrading agricultural value chains. It is assumed that it will make both these countries and their farmers better off. However, it is currently not well understood how the value created during value-addition activities is distributed along different levels in the value chain, what share of the value-added accrues to the farmers, and what are the possible constraints to participation in such value-addition activities. We look at this issue in the case of coffee in Ethiopia–its most important export product–and value-addition through ‘washing’ coffee, which is done in wet mills.

There are three major findings from our research. First, we find that washed coffee is being sold internationally with a substantial premium compared to ‘natural’ coffee and that this premium is largely transmitted to producers. However, while wet mills have become more widespread over time, only a minor share of Ethiopia’s coffee is exported as washed, and this share is not increasing over time. Even if coffee farmers have access to a wet mill, they often do not sell all their coffee cherries to them. Second, labor productivity for the production and marketing of red cherries, which is what is processed in wet mills, is significantly lower than for dried cherries. The greater labor required reduces the incentives for adoption of red cherry production for those households that have higher opportunity costs of labor. Third, only impatient–often smaller–farmers sell red cherries, as more patient ones use dried and storable cherries as a rewarding savings instrument, given the negative real deposit rates in formal savings institutions.

These results point to a number of potential policy implications for stimulate washing in Ethiopia’s coffee sector, a desire of the government given that the government could earn significantly higher export income by exporting more washed coffee. First, the Ethiopian government requires farmers not to sell directly to wet mills, but to go through primary marketing centers [38]. This inflates the costs for farmers in marketing red cherries. A review of this marketing requirements would be useful as it might bring down these marketing costs and improve relative profitability of red cherries. Second, we show the importance of labor demands in adoption decisions of improved production technologies. Support to research to develop labor-reducing innovations that could possibly accompany such technologies might be possibly useful areas to be further pursued. Third, high inflation, low or even negative deposit rates in formal banking institutions, and limited access to such institutions is often a typical characteristic of these economies. This often results in low formal savings rates, possibly hampering investments and growth [69]. However, missing formal savings markets do not imply that farmers do not save. Rather, they often rely on second-best mechanisms to overcome savings hurdles, as illustrated in this paper and elsewhere [32, 33]. Addressing these larger issues for the rural economy would possibly contribute to increased adoption of activities that contribute to higher value-addition for the country.

## Supporting information

S1 Fig Comparison of nominal coffee prices in major coffee producing zones, May (year t) versus November (year t-1), 2001 to 2016. Source: Authors’ calculation based on data from CSA’s retail price data.
(XLSX)
